# Characterization and expression profiling of microRNAs in response to plant feeding in two host-plant strains of the lepidopteran pest *Spodoptera frugiperda*

**DOI:** 10.1186/s12864-018-5119-6

**Published:** 2018-11-06

**Authors:** Yves Moné, Sandra Nhim, Sylvie Gimenez, Fabrice Legeai, Imène Seninet, Hugues Parrinello, Nicolas Nègre, Emmanuelle d’Alençon

**Affiliations:** 1 DGIMI, Univ Montpellier, INRA, Montpellier, France; 2 INRA, UMR Institut de Génétique, Environnement et Protection des Plantes (IGEPP), BioInformatics Platform for Agroecosystems Arthropods (BIPAA), Campus Beaulieu, Rennes, France; 3 INRIA, IRISA, Genscale, Campus Beaulieu, Rennes, France; 4 MGX, CNRS, INSERM, Univ Montpellier, Montpellier, France

**Keywords:** Adaptation, Phenotypic plasticity, Regulation of gene expression, Insect plant interaction, microRNAs

## Abstract

**Background:**

A change in the environment may impair development or survival of living organisms leading them to adapt to the change. The resulting adaptation trait may reverse, or become fixed in the population leading to evolution of species. Deciphering the molecular basis of adaptive traits can thus give evolutionary clues. In phytophagous insects, a change in host-plant range can lead to emergence of new species. Among them, *Spodoptera frugiperda* is a major agricultural lepidopteran pest consisting of two host-plant strains having diverged 3 MA, based on mitochondrial markers. In this paper, we address the role of microRNAs, important gene expression regulators, in response to host-plant change and in adaptive evolution.

**Results:**

Using small RNA sequencing, we characterized miRNA repertoires of the corn (C) and rice (R) strains of *S. frugiperda*, expressed during larval development on two different host-plants, corn and rice, in the frame of reciprocal transplant experiments. We provide evidence for 76 and 68 known miRNAs in C and R strains and 139 and 171 novel miRNAs. Based on read counts analysis, 34 of the microRNAs were differentially expressed in the C strain larvae fed on rice as compared to the C strain larvae fed on corn. Twenty one were differentially expressed on rice compared to corn in R strain. Nine were differentially expressed in the R strain compared to C strain when reared on corn. A similar ratio of microRNAs was differentially expressed between strains on rice. We could validate experimentally by QPCR, variation in expression of the most differentially expressed candidates. We used bioinformatics methods to determine the target mRNAs of known microRNAs. Comparison with the mRNA expression profile during similar reciprocal transplant experiment revealed potential mRNA targets of these host-plant regulated miRNAs.

**Conclusions:**

In the current study, we performed the first systematic analysis of miRNAs in Lepidopteran pests feeding on host-plants. We identified a set of the differentially expressed miRNAs that respond to the plant diet, or differ constitutively between the two host plant strains. Among the latter, the ones that are also deregulated in response to host-plant are molecular candidates underlying a complex adaptive trait.

**Electronic supplementary material:**

The online version of this article (10.1186/s12864-018-5119-6) contains supplementary material, which is available to authorized users.

## Background

Successful adaptation to the host-plant is of fundamental importance to herbivorous insects. It requires that the adult female accepts the host-plant for oviposition and that the host allows feeding and proper development of larvae. Understanding of the underlying genetic mechanisms used by insects in response to their host-plants ([1] for review) recently progressed thanks to availability of new reference genomes and transcriptomes of major polyphagous herbivorous insect pests. Genomic sequences analyses highlighted expansion of chemosensory and/or detoxification genes in generalist herbivores compared to specialist ones reflecting their larger diets [2–6]. RNA-seq analyses revealed that generalist herbivores use transcriptional plasticity of various categories of genes in response to their diet [3, 6–9]: detoxification, digestion, cuticular and ribosomal genes. Transcriptional plasticity of specific sets of genes has also been shown in an oliphagous lepidopteran species, *Manduca sexta*, when it is reared on host -or acceptable non-host plants [10]. These data show that a change of host-plant in herbivorous insect requires large scale transcriptional changes involving combinations of various gene family members.

While the nature and role of protein coding genes involved in adaptation to the host-plant begin to emerge, the putative role of non-coding genes has yet to be explored. Among them, microRNAs (miRNAs) are a class of small non coding RNAs (sncRNAs) of approximately 22 nt in length, which act as post-transcriptional regulators of gene expression and are known to help fine-tune complex genetic networks ([11], for review). The mode of action of miRNAs results in relatively weak modulation of less than twofold both at the RNA and protein levels [12]. Two other classes of small non coding RNAs combat the invasion and the expansion of transposable elements (TE), the short interfering RNA (siRNA) pathway suppresses TEs in all tissues of plant and animals, whereas the Piwi-interacting RNA pathway (piRNA) seems more specific to the gonads of metazoans (see [13] for review).

Since miRNAs have been shown to be involved in many physiological or cellular processes such as differentiation, proliferation, apoptosis and development [14], we hypothesized that they may play a role in adaptation of a phytophagous insect to its host-plants and that they may show different expression patterns in different host-plant races of the insect. To test this hypothesis, we used the noctuid moth *Spodoptera frugiperda*, which consists of two host-plant strains, one mostly associated to corn (C strain or SfC), the other to rice (R strain or SfR), and whose genomes are recently available [4]. We performed reciprocal transplant (RT) experiments of the two strains on the two host-plants and isolated and sequenced sncRNAs from feeding larvae. We present the differential expression patterns of miRNAs and their putative coding genes targets involved i) in phenotypic plasticity (the ability of a single genotype to produce multiple phenotypes in response to variation in the environment) of each strain in response to corn or rice ii) in adaptive evolution or genetic drift, by additional comparison of the two strains on the same host-plant.

## Results

### Deep sequencing of *S. frugiperda* small RNA

**Fig. 1:**
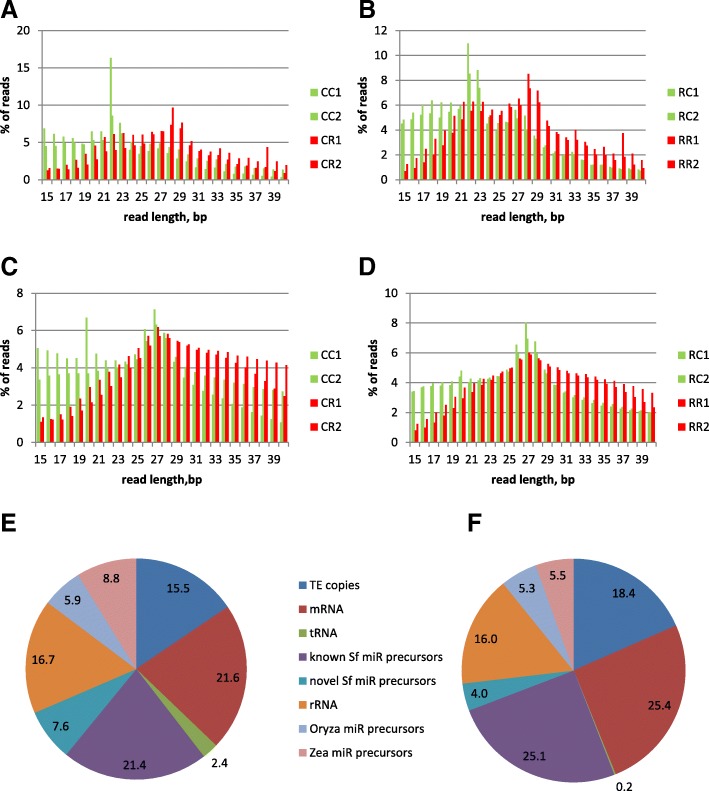
Size profiling of small non coding RNAs and their homology to different RNA classes or to Transposable Elements (TE). The percentage of sncRNA reads is plotted as a function of their size (between 15 nt to 40 nt corresponding to the size range that has been selected from the gel for library construction), **a** and **c** SfC, **c** and **d** SfR, in green on corn, in red on rice. CC: SfC on corn, CR: SfC on rice, RC: SfR on rice, RR: SfR on rice. **a** and **b** total reads, **c** and **d** unique reads. **e** and **f** Pie charts representing the average % of reads (total counts from 2 replicates on corn for SfC (**e**) or SfR (**f**)) mapping either to SfC or plant miR precursors, or TE (SfC TE copies) as expected for putative endo-siRNA or piRNAs, or mRNA (SfC OGS2.2), or SfC tRNA, SfC rRNA (18S and 28S RNAs)

To characterize *S. frugiperda* miRNA, small RNA libraries were constructed from whole body of corn-fed and rice-fed larvae of *S. frugiperda* C and R strains. Two independent libraries (biological replicates) were prepared and independently sequenced using Illumina technology. Between 31.5 to 57 millions high quality sequence reads were obtained after adapter trimming in each library (See Additional file 1: Table S1). Their size distribution shows an over-abundance of sequences at 22 nt, typical of miRNAs (Fig. 1a-b). On average, we detected 11.12+/− 3.67% of sequences of size 22 nt on corn and 5.49+/− 1.06% on rice. We detected a second peak between 25 and 33 nt that could correspond to expression of piRNAs. After collapsing the reads, we identified between 2 to 5 million unique sequences, 4% of which (4.2+/− 0.2% on corn and 3.71+/− 0.52% on rice) on average corresponding to miRNAs of 22 nt (Fig. 1c, d). However, in term of diversity, piRNAs seem more abundant than miRNAs (Fig. 1c, d) which may reflect abundance and diversity of transposable elements (TE) in the genome of *S. frugiperda* (29.14% and 29.10% of genome coverage by TE in C and R strain, respectively [4]).

In eukaryotic cells, the vast majority of cellular RNA consists of ribosomal RNA (rRNA) - ~ 80 to 90% of total RNA for most cells, followed by transfer RNA (tRNA) - 10 to 15%, messenger RNA (mRNA) - 3 to 7%, miRNA - 0.003 to 0.02% [15]. The small ncRNA sequences of SfC or SfR were aligned against these different references, in addition to the sequences of TE copies, which should highlight putative piRNAs or endo-siRNAs (Fig. 1e, f), The most abundant hits (29–29.1%) were found matching to Sf miRNAs precursors (precursors of known or novel miRNAs sequences) or Sf TE copies (15.5–18.4%) corresponding to putative piRNAs or endo-siRNAs. 16–16.7% of them matched to rRNA, and 0.2–2.4% to tRNA, the most abundant RNA classes expected in the cells. These data show that the sRNA sequences were enriched in functional miRNAs or TE-interacting RNAs compared to those resulting from degradation of ribosomal or transfer RNAs. We found also sncRNA sequences matching to miRNA precursors of plants (*Zea* or *Oryza*). It is expected since sncRNAs have been extracted from whole larvae feeding on plants. The lack of sequence homology between miRNA families in plants and animals (with exception of one family [16]) as well as differences in their biogenesis and mode of action suggested that miRNAs have evolved independently in both kingdoms from an ancient siRNA mechanism already present in the last common ancestor of all eukaryotes [12]. The presence of plant miRNA reads in larval samples is thus not expected to interfere with the study of animal miRNAs performed in this paper.

### Annotation of *S. frugiperda* miRNA genes

#### miRDeep2 analysis

To detect miRNA genes, raw sequencing data were analyzed with miRDeep2 software. miRDeep2 [17, 18] maps the sRNA reads to the genome and excises potential miRNA precursors sequences from the genome. The secondary structures of the miRNA precursors are predicted and their stability is estimated by RNAfold. mirDeep2 uses a probabilistic model of miRNA biogenesis by the Dicer protein to score frequency and compatibility of mapping of the small RNA sequence reads (the signature) on the secondary structure of the miRNA genomic precursors (the structure) as compared to a non-miRNA precursor hairpin [17, 18]. Read stacks correspond to mature miRNA sequences. The score reflects the likelihood of each precursor to be a genuine miRNA. Furthermore, since the algorithm may generate hairpins with read stacks that have no connection with miRNA biology, corresponding to false positives, miRDeep2 estimates the rate of false positive by shuffling the observed combinations of structures and signatures and submit them to the core algorithm. The difference in score distribution between the genuine combinations and the control ones is used to estimate the number of false positive novel miRNAs for varying score cut-offs. The sequence of mature predicted miRNAs are compared to mature miRNA sequences contained in miRBase (release 21) which allows to sort them in two classes, known or novel depending if they existed or not in miRBase. The genomes of the C strain (v3.1) and the R strain (v1.0) were used as references using a pool of sequence reads from either the C or the R strain, respectively. As shown on Table 1, we obtained 76 and 68 known miRNAs predicted genes (70 and 64 unique ones) and 139 and 171 novel ones (126 and 158 unique ones) in the C and R strain.

**Table 1:** Number of miRNAs genes predicted in the *Spodoptera frugiperda* genome

Species	Known	New compared to Kakumani^a^	Known and unique	Known Score > 4 (+randfold yes)	Novel	New compared to Kakumani^a^	Novel And unique	Novel Score > 4 (+randfold yes)
SfC	76	40	68	66 (66)	139	103	126	92 (78)
SfR	68	36	64	61 (59)	171	155	158	115 (102)

^a^Since the *S. frugiperda* microRNAs predicted by Kakumani et al., [21] are not registered in miRBase, we checked whether each of our predicted miRs had been predicted in Sf21 genome

We calculated for the 139 novel miRs predicted in SfC, the processing precision frequency, defined as the ratio of reads corresponding exactly to the mature miRNA and miRNA* sequences, divided by the total amount of reads mapping to the hairpin [19] (See Additional file 2: Excel file S1, tabs “SfC Novel”). A value close to one indicates high precision and a value close to zero indicates the production of very few miRNA/miRNA* duplexes, with a cut-off of less than 0.1 corresponding to low processing precision [20]. One hundred thirty six novel miRs (97.8%) have an efficiency of more than 0.1, and 111 (79.8%) have an efficiency of > 0.5 thereby showing a medium to high processing precision in *S. frugiperda* and*/or that the mirDEEP2 prediction generated few false positives.

After filtering for the prediction showing a score > 4 (the lowest score cut-off corresponding to a prediction signal-to-noise ratio *r* > 10, r = total miRNA hairpins reported/ mean estimated false positive miRNA hairpin over 100 rounds of permutated control) and a significant randfold *p*-value, we obtained 66 and 59 genes for known miRNAs and 78 and 102 for novel ones in C and in R strain. All miRNA genes prediction can be found in Supplementary excel file 1 (See Additional file 2). Compared to the miRNAs identified from ovary cell lines of *S. frugiperda* (*Sf*21) [21] whose sequences are not in the miRBase release 21, we found 40 to 36 additional predictions of known miRNAs genes and 103 to 155 novel ones in C and R strain.

Other miRs involved in response to Baculovirus infection have been described from another ovary cell lines of *S. frugiperda, Sf*9, with precursor sequences mapped on *Bombyx mori* genome [22].

**Fig. 2:**
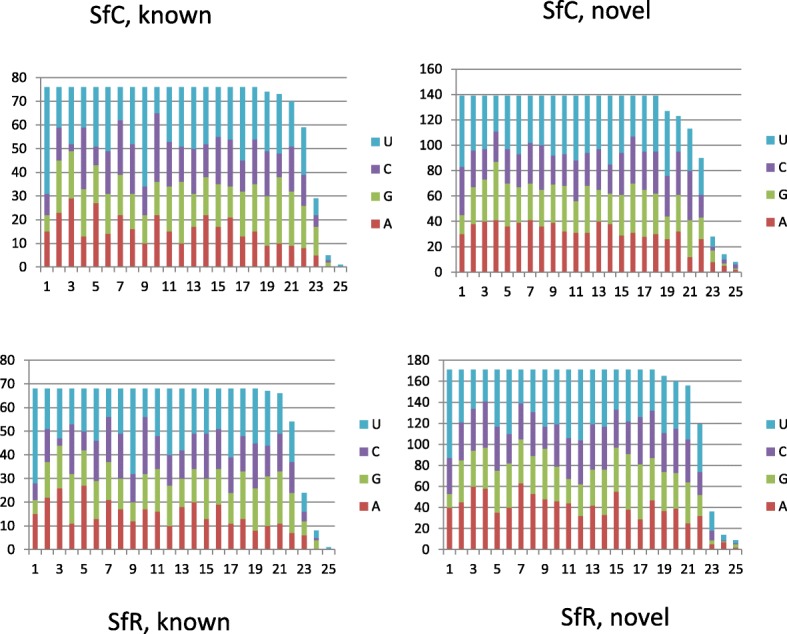
Base composition of known or novel mature miRNAs

The specific nucleotide occurrence was analyzed in the obtained miRNA sequences, *S. frugiperda* showed a dominant bias for uracil (U) at the first nucleotide (Fig. 2). The dominance of U at first position towards 5’end is a conserved feature of miRNAs [23].

#### Orthology

To identify orthologous miRNAs between C and R strain a reciprocal blastn was performed between mature sequences of known or novel miRNAs at an e-value < 0.001. We identified 57 orthologs among known miRNAs and 75 among novel ones. The list of orthologs can be found in Additional file 2: Excel file S1. This step allowed comparison of expression levels of miRNAs according to the genetic background (C or R strain).

### miRNA expression variation

#### DESeq2 analysis according to host-plant

Expression values generated by miRDeep2 in the RT experiments were used to analyze differential expression of *S. frugiperda* miRNAs according to the host-plants in each strain. In Additional file 3: Figure S1, for SfC in the upper part and SfR in the lower part, the MA-plots show the log2 fold change on rice versus corn over the mean of normalized counts, i.e. the average of counts normalized by size factors. The points with FDR less than 0.05 are colored in red. We also visualized samples (treatments, replicates) by Principal Component Analysis (PCA) as shown in Additional file 4: Figure S2. Most variation was linked to treatment, i.e., change of host-plant, the smallest variation was found between biological replicates.

**Fig. 3:**
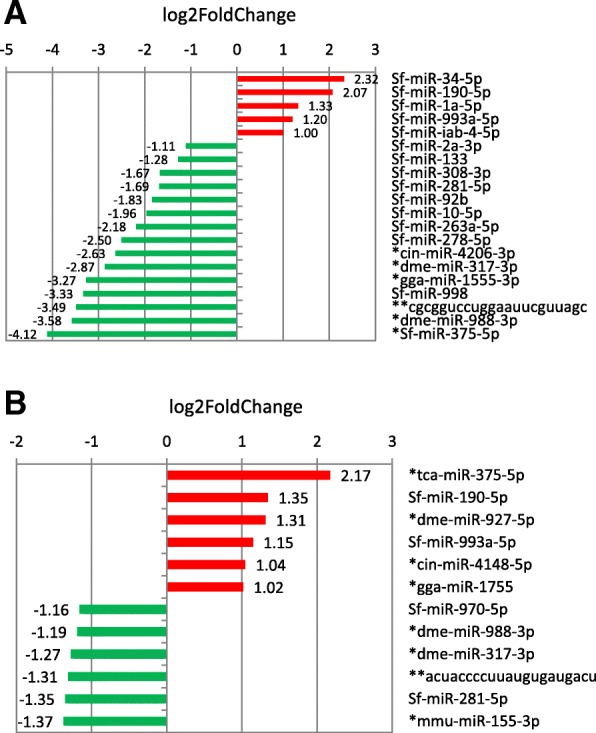
Differential expression of miRNAs genes on rice compared to corn in *Spodoptera frugiperda* larvae (L4 instar), after rearing for 3 generations on whole plants. The miRNAs showing a significant differential expression after DESeq2 analysis (log2foldchange > 1 or < 1 and FDR < 0.05) are shown. **a** In SfC **b** In SfR. In red, miRNAs up-regulated on rice, in green miRNAs up-regulated on corn

Based on read counts, 34 known or novel miRNAs (out of 144, 23.6%) were differentially expressed (FDR < 0.05) in the C strain larvae fed on rice as compared to the C strain larvae fed on corn (Details of DESeq2 analyses can be found in Additional file 5: Excel file S2.Twenty one known or novel miRNAs (out of 161, 13%) were differentially expressed on rice compared to corn in R strain. For example, in the C strain, known miR-34-5p and miR-190-5p were overexpressed on rice (in red on Fig. 3a), while novel miR-375-5p was overexpressed on corn (in green). In the R strain (Fig. 3b), novel tca-miR-375-5p and known miR-190-5p were overexpressed on rice as compared to corn (in red). Novel mmu-miR-155-3p was overexpressed on corn compared to rice (in green). We used TaqMan RT-qPCR miRNA assays on total RNA extracted from the RT experiments to validate expression variation based on read counts, for some of the most differentially expressed miRNAs (Table 2). We could confirm that known miR-34-5p and miR-190-5p were significantly overexpressed on rice compared to corn in the C strain and that known miR-190-5p and novel tca-miR-375-5p were significantly overexpressed on rice as compared to corn in the R strain (Table 2).

**Table 2:** Experimental validation of variation in miRNA expression

Experimental condition	MicroRNA	Relative expression	Std Error	95% C.I.	P(H1)	Result
SfC (Rice/Corn)	miR-31 (Ref)	1				
	miR-34	1.763	(1.080–3.062)	1.003–3.513	0.004	UP
	miR-190	1.549	(1.320–1825)	1.235–2.032	0.001	UP
SfR (Rice/Corn)	miR-31 (Ref)	1				
	miR-190	1.218	0.671–1.520	0.900–1.669	0.05	UP
	tca-miR375*	5.802	(3.115–11,105)	1.511–14.148	0.002	UP
On Corn (SfR/SfC)	miR-31(Ref)	1				
	miR-34	1.868	1.395–2.466	1.282–2.811	0.003	UP
	dme-miR-275*	0.965	0.821–1.121	0.726–1.251	0.587	
On Rice (SfR/SfC)	miR-31(Ref)	1				
	dme-miR-275*	1.434	1.236–1.545	1.194–1.907	0.001	UP

The relative expression of miR genes depending on the host-plant (on rice compared to corn) in either the C or the R strain, or depending on the genetic background (SfR compared to SfC) was calculated according to [57]

*P(H1)* Probability of alternate hypothesis that difference between sample and control groups is due only to chance. The miRs with a star correspond to * Novel miRs having conserved seed region only

#### miRNA expression variation between strains

We compared expression values between the two strains when they were reared on the same host plant to detect differences linked to the genetic background. In Additional file 6: Figure S3, on corn (top panel) and on rice (bottom panel), the MA-plots show the log2 fold change in SfR versus SfC over the mean of normalized counts. Again by PCA analysis of samples, more variation was found between strains than between biological replicates (See Additional file 7: Figure S4).

Based on reads counts, nine known or novel miRNAs (out of 129, 6.97%) were differentially expressed (FDR < 0.05) in the R strain compared to C strain when reared on corn (See Additional file 8: Excel file S3). A similar number of miRNAs (10 out of 129, 7.75%) were differentially expressed between strains on rice.

**Fig. 4:**
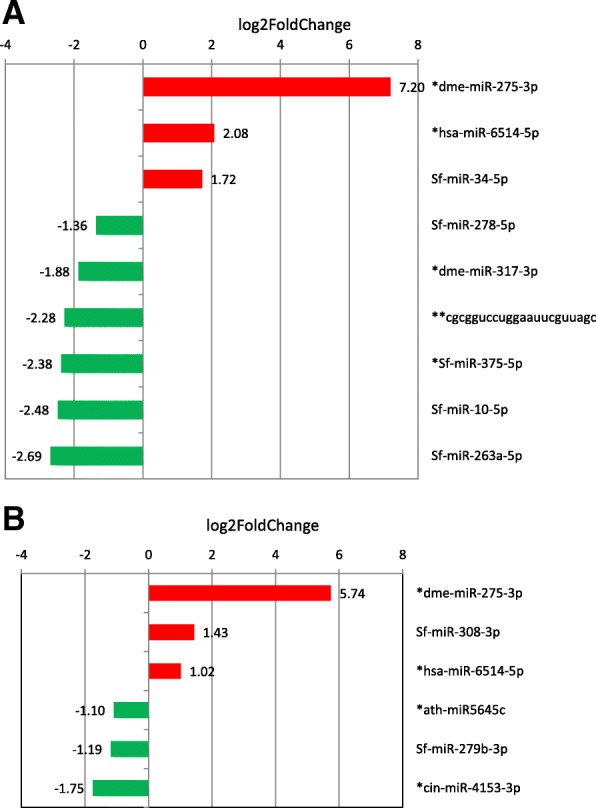
Differential expression of miRNAs genes according to the genetic background. The relative expression of miRNAs in SfR compared to SfC is shown, either on corn (**a**) or on rice (**b**). In red, miRNAs up-regulated in SfR, in green miRNAs up-regulated in SfC

For example, known miR-34-5p and novel dme-miR-275-3p were overexpressed in SfR compared to SfC on corn (in red on top of Fig. 4), the latter being also overexpressed in SfR compared to SfC on rice (in red on bottom of Fig. 4). Using TaqMan RT-qPCR miRNA assays, we could confirm upregulation of miR-34-5p in SfR compared to SfC on corn, and that of the novel dme-miR-275-3p in SfR compared to SfC on rice (Table 2), however not on corn, maybe due to the presence of a RNA molecule competing with the microRNA for the TaqMan probe in this condition.

#### Expression differences both between strains and between plants

**Fig. 5:**
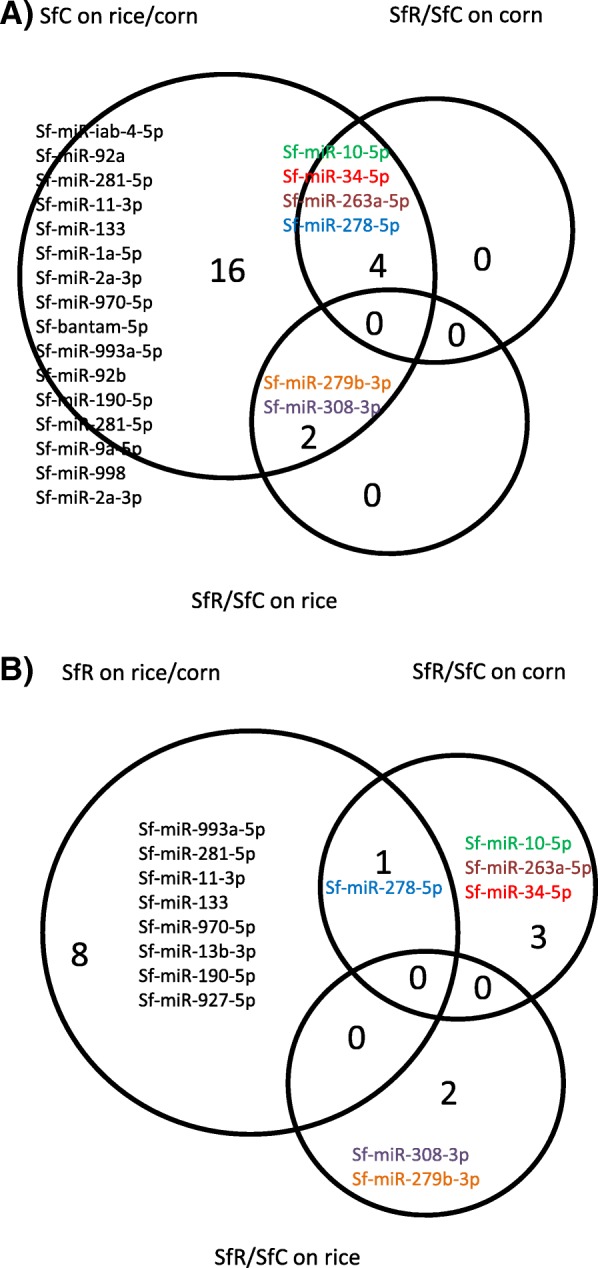
Are the constitutive expression differences between strains involved in phenotypic plasticity within strains? This Venn diagram highlights the miRNAs that are differentially expressed (FDR < 0.05) both between strains on the same plant and within strain (SfC: (**a**), SfR: (**b**)) on rice compared to corn

The expression differences between host-strains may result from genetic drift and also possibly from divergent selection by the environment, in this case the different host-plants. To identify the ones putatively involved in adaptation to the host-plant, we looked for the miRNA genes of the known class that were differentially expressed both between strains and between plants (FDR < 0.05). As shown on Fig. 5a, four miRs (miR-10-5p, miR-34-5p, miR-263a-5p, miR278-5p) were differentially expressed both constitutively between strains on corn, and within SfC when reared on different plants. The two miRNAs genes that were differentially expressed between strains on rice (miR-279b-3p and miR-308-3p) were also differentially expressed in SfC on different plants. On Fig. 5b, we found that among the 4 miRNAs that were differentially expressed between SfR and SfC on corn, only one, miR-278-5p, was also differentially expressed when SfR was reared on rice compared to corn. None of the two miRNAs that were differentially expressed between strains on rice was differentially expressed in SfR according to the host-plant. Most constitutive differences between strains are involved in interaction with the plant in SfC, less of them in SfR, suggesting that adaptation to the plant played a more pronounced role in SfC evolution than in SfR.

### Potential target genes regulated by miRNA

mRNA targets of known miRNAs were searched using TargetScan, miRanda, Rna22 and miRmap (See Methods) against the 3’ UTR of the C strain gene set, OGS2.2. The target gene list of the seven DE miRNAs identified in this study (miR-34, miR-190, miR-1a-5p, miR-998, miR-278, miR-263a-5p, miR-10-5p) can be found in Additional file 9: Excel file S4. Among the gene targets predicted by TargetScan, we filtered out those showing variation in their expression in the RT experiments (according to [7]). We also checked whether they were predicted as miR targets by at least one of the three other softwares. The list of target genes that are differentially expressed in SfC on different host plants can be found in Additional file 10: Excel file S5. The target genes that are differentially expressed in SfR compared to SfC on corn are listed in Additional file 11: Excel file S6.

### Potential regulated target genes

To reduce the number of putative false positive coding gene targets among those predicted by TargetScan, we assumed that true targets should be expressed in the same experimental conditions as miRNA genes, and downregulated when miRNA genes were overexpressed. To identify these candidate genes, we used RNA-Seq results obtained in the same reciprocal transplant experiments after two generations on plants [7]. We limited the analysis of target genes to the Official Gene Set of the C strain, which is the reference and has been manually curated [4], and to RT experimental conditions in which the RNA-Seq data of putative target genes were available in duplicates in [7]. We thus provide a detailed analysis of targets genes in the following conditions: 1) SfC reared on corn and on rice 2) SfC and SfR when reared on corn. We focused on the most differentially expressed miRNAs of “known” class (FDR < 0.05 and absolute value of log2 fold change > 1.3) in these conditions. The complete list of differentially expressed (FDR < 0.05) coding gene targets of these known miRNA genes, with their log2 fold change and annotation can be found in Additional file 10: Excel file S5 and Additional file 11: Excel file S6. MiR-34 and miR-190 are up-regulated in SfC when reared on rice, a non-host plant. Among down-regulated targets of miR-34, we found members of different gene families (Table 3), some of which are also targets of miR-190: First, a representative of the takeout gene family, then three genes encoding cuticle proteins. A gene encoding a subunit of the 26S proteasome was specifically targeted by miR34. As miR-190 specific targets, were found a cuticle protein gene, an acyl-CoA desaturase gene, and a member of the Osiris gene family, *osi9a* [7].

**Table 3:** List of target genes of known miRs and their differential expression

MiR				Putative target genes				
Name	Sf strain	Regulated on	Log2FC	^a^Predicted Targets	Down or up regulated targets	Down-regulated	^b^Among down and up- regulated genes	Log2FC
miR-34			2.32	321	38	28	take-out GSSPFG00021718001-RA cuticular proteins	−4.22
							GSSPFG00000626001-RA	−3.24
							GSSPFG00013845001-RA	−1.6
							GSSPFG00010502001-RA subunit DSS1 of 26S proteasome	−1.21
							GSSPFG00027010001-RA	−2.14
miR-190			2.07	479	46	25	take-out GSSPFG00021718001-RA cuticular protein	−4.22
							GSSPFG00005155001-RA acyl-CoA desaturase	−3.54
							GSSPFG00006314001-RA osiris 9A	−6.54
							GSSPFG00012033001-RA	−4.71
miR-1a-5p			1.33	524	46	18	Serine proteinase GSSPFG00014426001-RA	−3.38
miR-998			− 3.33	229	31	10	sodium- and chloride-dependent glycine transporter 1-like GSSPFG00018036001-RA facilitated trehalose transporter Tret1	2.61
							GSSPFG00023681001-RA Spermine oxidase	1.31
							GSSPFG00025432001-RA	3.02
miR-278			−2.50	91	8	4	integrin alpha-PS2 GSSPFG00025987001	1.15
miR-263a-5p			−2.18	436	56	27	sodium- and chloride-dependent glycine transporter 1-like GSSPFG00018036001-RA facilitated trehalose transporter Tret1	2.61
							GSSPFG00023681001-RA Spermine oxidase	1.31
							GSSPFG00025432001-RA	3.02
miR-10-5p			−1.96	151	20	12	sodium- and chloride-dependent glycine transporter 1-like GSSPFG00018036001-RA facilitated trehalose transporter Tret1	2.6
							GSSPFG00012081001-RA	1.61
Name	Host-plant	Regulated in	Log2FC	^a^Predicted Targets	Down and up- regulated targets	Down-regulated	^b^Among down and up- regulated genes	Log2FC
miR-34			1.72	321	30	18	transcription factor ap-2 GSSPFG00001565001-RA lysine-specific demethylase 2B	−1.3
							GSSPFG00019875001-RA graves disease carrier protein	−1.24
							GSSPFG00028641001-RA	−1.08
miR-263a-5p			−2.69	436	43	26	unknown secreted protein [Papilio polytes] GSSPFG00016698001-RA cuticular protein RR-1 motif 26	1.96
							GSSPFG00021712001-RA	1.54
miR-10-5p			−2.48	151	25	7	proton-coupled folate transporter GSSPFG00020391001-RA facilitated trehalose transporter Tret1-like	1.11
							GSSPFG00012081001-RA	1.02
miR-278-5p			−1.36	91	7	3	zinc finger protein 347 GSSPFG00017383001-RA	0.76

Clipart for rice is available by Google [Apache License 2.0 (http://www.apache.org/licenses/LICENSE-2.0)], via Wikimedia Commons, https://upload.wikimedia.org/wikipedia/commons/5/58/Emoji_u1f33e.svg and for corn by Spedona - Own work, CC BY-SA 3.0, https://commons.wikimedia.org/w/index.php?curid=5310440

^a^Target genes were predicted using Targetscan software. ^b^The predictions that had been confirmed by at least one other software among miRanda, Rna22 or miRmap and that had been shown to be expressed and regulated in parallel Reciprocal Transplant experiments [7] are listed with the corresponding foldchange. The complete target gene list figure in Additional file 10: Excel file S5 and Additional file 11: Excel file S6. Pictures of *S. frugiperda* larvae on plants were taken by Marion Orsucci who gave the written permission to use and adapt them

Among miRNAs that are overexpressed in SfC on corn compared to rice, three, miR-998, miR-263a-5p and miR-10-5p share two targets that encode transporters (facilitated trehalose transporter Tret1 and a sodium- and of chloride-dependent glycine transporter 1) that are downregulated on corn. In addition, a gene encoding a spermine oxidase enzyme is the target of both miR-998 and miR-263a-5p.

When we compared the two strains on the same host-plant corn (See Additional file 11: Excel file S6), we found that miR-34 was up-regulated in SfR and that miR-263a-5p, miR10-5p and miR-278-5p were up-regulated in SfC. Among the down-regulated targets of miR-34, we found a representative of the transcription factor family AP-2 (Table 3). Among the down-regulated targets of miR-263a-5p, were found a protein of unknown function and a cuticular protein. Among the targets of miR10-5p, two genes coding for transporters (proton-coupled folate transporter, facilitated trehalose transporter Tret1) were found.

**Fig. 6:**
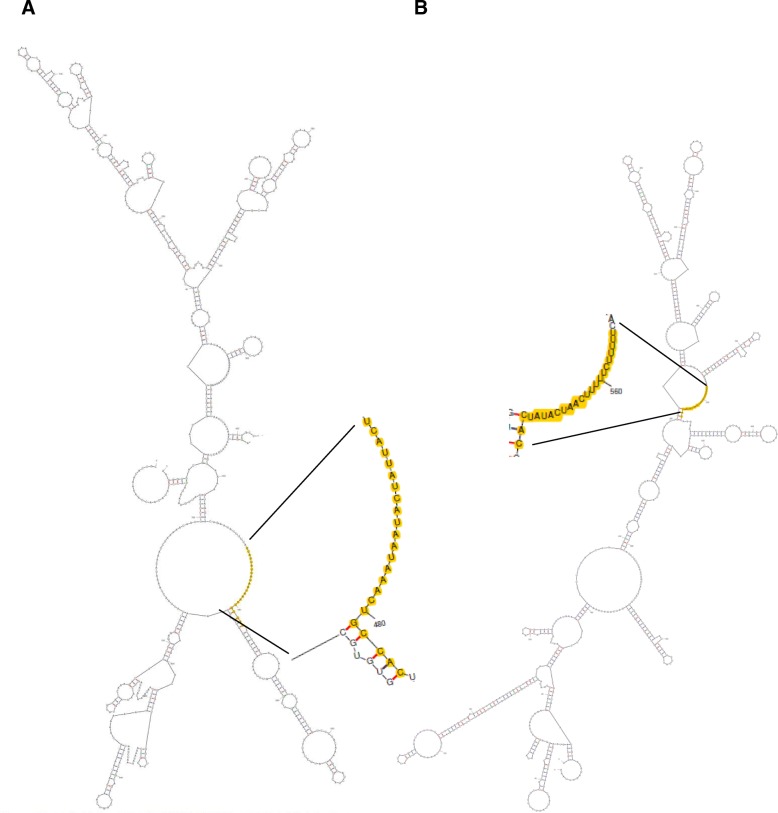
Examples of complementarity between miRNAs seed sequences and the secondary structure of their putative targets. Using mfold [25] to predict and draw secondary structures of 3’UTR of the predicted targets, we could show that miR-34 and miR-190 map to loops rather than stems in the secondary structures of their targets, takeout (GSSPFG00021718001-RA) in (**a**) and acyl-CoA desaturase (GSSPFG00006314001-RA) in (**b**), their most differentially expressed candidates, respectively

Because of the energy required for freeing base-pairing interactions within mRNA in order to allow miRNA binding, secondary structures have been shown to contribute to target recognition by miRNAs [24]. Using mfold [25] to predict secondary structures of 3’UTR of predicted targets, we could show that miR-34 and miR-190 map to loops rather than stems in the secondary structures of their targets, takeout (GSSPFG00021718001-RA) and acyl-CoA desaturase (GSSPFG00006314001-RA), their most differentially expressed candidates respectively (Fig. 6a and b), which may facilitate the interaction.

## Discussion

In another paper [7], we have shown that the two host-plant strains of *Spodoptera frugiperda* show phenotypic and transcriptional plasticity when reared on their preferred versus alternative host-plants, corn or rice. In this paper, using similar reciprocal transplant experiments, we have shown variation in expression profile of miRNAs in the two lineages according to the host plant or between lineages on the same host-plant, either corn or rice. We identified putative targets of differentially expressed miRNAs in the 3’UTR of coding genes, and present a detailed analysis of those that are expressed i) in the same experimental condition as miRNAs ii) in the opposite direction (downregulated when miRNAs are upregulated and vice versa), that we consider the most reliable candidates.

Among down-regulated targets of miR-34, we found (See Additional file 10: Excel file S5, Table 3) members of different gene families, some of which are also targets of miR-190: First, a representative of the takeout gene family, putatively involved in feeding behavior and response to starvation as in *D. melanogaster* [26]. This representative is targeted both by miR-34 and miR-190. Second, three genes encoding cuticle proteins whose downregulation may reflect slower development of SfC on rice compared to corn [7]. Another cuticle component is also targeted by miR-190. Among miR-34 specific targets, we found a gene encoding a subunit of the 26S proteasome, which can be involved in protein degradation in response to oxidative stress. In the case of phytophagous insects, oxidative stress can be generated by prooxidant allelochemicals produced by host-plants. Among miR-190 specific targets, an acyl-CoA desaturase gene, necessary for fatty acid biosynthesis, may be repressed because rice is a poor food for SfC, and a member (*Osiris 9*) of the Osiris gene family, putatively involved in response to plant toxins as in *Drosophila sechellia* [27]. A recent analysis of the conserved patterns of Osiris gene expression in different insect species, suggests that Osiris genes may play a central role in insect adaptive evolution [28].

The three miRNAs miR-998, miR-263a-5p and miR-10-5p that are overexpressed in SfC on corn share two targets that encode transporters (facilitated trehalose transporter Tret1 and a sodium- and of chloride-dependent glycine transporter 1) that are downregulated on corn (up-regulated on rice). In most insects, trehalose (a-D-glucopyranosyl-(1,1)-a-D-glucopyranoside) is the main haemolymph sugar. In Drosophila, Tret 1 is necessary for the transport of trehalose produced in the fat body and its uptake into other tissues that require a carbon source, and thereby regulates trehalose levels in the hemolymph [29]. The glycine transporter GLYT1, by controlling the reuptake of glycine at synapses [30], regulates neurotransmission, where glycine plays the role of inhibitory neurotransmitter. In addition a gene encoding a spermine oxidase enzyme is the target of both miR-998 and miR-263a-5p. Polyamines (PA), comprising spermine (Spm), spermidine (Spd) and putrescine (Put), are ubiquitous polybasic molecules, with many important biological functions, like cell growth, differentiation, and apoptosis. They interact reversibly with nucleic acids, regulating chromatin status and gene expression, and modulating ion-channels’ function and stability (reviewed in [31]). Polyamine oxidases (PAO) include spermine oxidase. These enzymes, containing a flavin adenine dinucleotide (FAD), catalyze the oxidation of polyamines, and lead to formation of hydrogen peroxide. They regulate cellular polyamine concentration.

When we compared the two strains on the same host-plant corn (See Additional file 11: Excel file S6), we found that miR-34 was up-regulated in SfR and that miR-263a-5p, miR10-5p and miR-278-5p were up-regulated in SfC. Among the down-regulated targets of miR-34, we found a representative of the transcription factor family AP-2. From [32], AP-2 is expressed during Drosophila embryogenesis in the maxillary segment and neural structures, whereas during larval development, it is expressed in the central nervous system (CNS) and the leg, antennal and labial imaginal disks [33, 34]. In a Drosophila AP-2 mutant, defects in proboscis development and leg-joint formation have been described [35, 36]. We found a homolog of the graves disease carrier protein, a protein of as yet uncharacterized function that belongs to the mitochondrial metabolite carrier family (which includes the ADP/ATP translocator, the phosphate carrier and the hydrogen ion uncoupling protein). Among the down-regulated targets of miR-263a-5p, were found a protein of unknown function and a cuticular protein. Among the down-regulated targets of miR-10-5p, two genes coding for transporters (proton-coupled folate transporter, facilitated trehalose transporter Tret1) were identified. Folates are a family of B9 vitamins found in nature primarily as 5-methyltetrahydrofolate (5-methylTHF). 5-MethylTHF provides the methyl group for the synthesis of methionine from homocysteine and therefore is necessary for the formation of S-adenosylmethionine, which is required for a variety of methylation reactions [37]. In addition, folates are the sources of a methylene moiety for the de novo synthesis of thymidylate from deoxyuridylate and two formate moieties for the de novo synthesis of the purine ring. PCFT has been extensively studied in humans due to the importance of folates in cancer progression, however it has not been studied in insects. In humans, PCFT is expressed at the acidic microenvironment of the apical brush-border membrane of the proximal small intestine and allows the intestinal absorption of folates. In humans and mice, loss of function mutations in PCFT, results in severe systemic folate deficiency with anemia, sometimes pancytopenia, hypo-immunoglobulinemia and gastrointestinal defects [38].

This analysis of putative targets of DE miRNA reveals that they control important pathways necessary for cellular or organismal homeostasis during insect-plant interaction. Although we provide transcriptomic evidence of downregulation of putative targets, their experimental validation will require additional efforts like the use of biotin tagged miRNAs to capture them and/or translation profiling [39, 40], which will be the focus of future work.

For expression analysis of the predicted target genes, insect samples were collected after two generations on plants whereas for the study of miRNA expression they were collected after three generations. This was to ensure dilution of miRNAs putatively maternally inherited (it is the case for miR-34 in *D. melanogaster* for instance [41]) and related to the artificial diet on which the insect fed before the reciprocal transplant experiment on plants started. We considered that in controlled experimental conditions of growth, gene expression during each successive insect developmental cycle is reproducible and comparable from one generation to another (Since *S. frugiperda* is a quarantine organism in France, we performed the RT experiment in large incubators with controlled hygrometry, temperature and light conditions). If the plant exerted a selection pressure, gene allele frequencies may have changed but not significantly between two generations since the laboratory strain that we used has a limited genetic polymorphism.

For differential expression analysis of miR genes and their targets, two biological replicates of the reciprocal transplant experiment have been performed. Since the first use of RNA-Seq to analyze transcriptomic data [42], we know more on the parameters necessary to optimize detection of differentially expressed genes. Lamarre et al. recently showed that the read depth in part compensates for the number of replicates to increase the ratio of differentially expressed genes detected [43]: with 20 million reads (for 20,000 DE genes - corresponding to a coverage of 1000 reads per gene) and 2 replicates, 85% of DE genes are found, and 8 replicates are needed to reach a ratio of 100%, while with 2.5 million reads (~ coverage 125 reads/gene) and 2 replicates, only 15% of DE genes are detected and the use of 8 replicates increases the ratio of DE genes to only 60%. In our miR differential expression analysis, we performed only two biological replicates. However, the read depth in each SfC library (Additional file 1: Table S1) was 42 million on average, with more than 20% of the sequences having the expected size for miRs (21 to 23 nt), this corresponds to an average coverage of > 39,688 reads per miR gene, a higher coverage than the maximal one used in the study of Lamarre et al.. Lamarre et al. also showed that the rate of false positives obtained with DESeq2 is minimal with 2 replicates and increases with the number or replicates. According to them, 70% of true positives can be detected with two replicates and this number increases with the number of replicates. We conclude that our experimental design enabled detection of a reasonable number of reliable DE miR candidates although more repetitions may be necessary to deepen the study.

In the same line, our differential expression analysis was done from RNA extracted from whole body of the larvae feeding on plants. We are aware that this experimental design may underestimate the number of miRs showing variation in their expression due to the fact that overexpression in one tissue may be masked by down regulation in another one. It will be the subject of future effort to look for expression variation tissue by tissue.

The miRNAs expression differences that we uncovered between the host-strains may result from genetic drift and also possibly from divergent selection by the environment, in this case the different host-plants. To identify the latter, we searched for the miRNAs that were differentially expressed both between strains on the same plant and within strains in response to the host-plant. Interestingly, we found that all the miRNAs expression difference between strains on plants (6/6, miR-10-5p, miR-34-5p, miR-263a-5p, miR-278-5p, miR279b-3p, miR-308-3p) were also involved in response to the host-plant in SfC. By contrast, one miRNA only (miR-278-5p) out of the six that showed constitutive difference between strains, showed also variation in response to the host-plant in SfR. By measuring the fitness of the insects on plants, we had found better survival of the C strain on corn compared to rice [7] suggesting that SfC was adapted to corn. The miRNAs that are both deregulated between strains and in response to plant may be involved in this adaptive evolution of SfC.

MiRNAs have been shown to be involved in response to various environmental changes, like response to starvation in *C. elegans* [44], to freezing and anoxia stress in the freeze tolerant fly *Eurosta solidaginis* [45], to thermal plasticity of the Senegalese sole [46], to drought in Tobacco [47]. In the case of *Spodoptera frugiperda*, by regulating phenotypic plasticity, miRNAs may have also played a role in evolutionary adaptation as has been discussed in the case of human miRNAs [48]. Further work is needed to show that the miR candidates identified in this study are directly involved in fitness of the insect on its host-plant, however the possibility of transgenerational inheritance of these molecules in male or female gametes [41, 49, 50] suggests that they could facilitate transmission of complex adaptive traits from parents to offspring.

## Conclusions

In the current study, we performed the first systematic analysis of miRNAs in Lepidopteran pests reared on whole host and non-host plants. We identified a set of the differentially expressed miRNAs that respond to the plant diet, or differ constitutively between the two host plant strains. The analysis of the putative targets of these DE miRNAs revealed that they control important pathways necessary for cellular or organismal homeostasis during insect-plant interaction. Since two classes of non coding RNAs, siRNAs and miRNAs have been used to control insect development [51, 52], this study of regulatory molecules of insect - plant interaction can bring clues on novel environment friendly biological control of crop pests.

## Methods

### Biological material

Two laboratory strains of *S. frugiperda* were used in this study: the corn-strain, originated from French Guadeloupe and the rice-strain, originated from Florida, USA. Each strain was reared on its principal and alternative host plant (*Zea mays* L. cv B73 or *Oryza sativa L. japonica* cv Arelate) under controlled conditions (temperature: 24 °C, photoperiod 16:8 light:dark, relative humidity: 65%). Eggs from *S. frugiperda* were deposited on plant leaves and fed ad libitum. After three generations on plants, larvae of fourth instars (L4) were collected. Therefore, larvae selected for small RNA extraction and sequencing comprised four groups: C strain reared on corn (CC), R strain reared on corn (RC), C strain reared on rice (CR) and R strain reared on rice (RR). Each experiment comprised two independent replicates.

### Small RNA extraction and sequencing

Small RNA was extracted from 12 L4 larvae using the mirVana miRNA Isolation Kit (Ambion) according to the manufacturer’s instructions. Extracted small RNA was quantified using a NanoDrop 2000 spectrophotometer (Thermo Scientific) and analyzed by PAGE and silver-staining. Small RNA samples from each replicate of CC, RC, CR and RR larvae were used for cDNA library preparation using the TruSeq ®Small RNA Sample Prep Kit (Illumina) and sequencing on Illumina HiSeq 2500. A size selection targeting snRNAs in the range of 15 to 40 nt was done. Library preparation and sequencing were performed by the platform MGX-Montpellier GenomiX (Montpellier, France).

Computational analysis of small RNA sequencing data and miRNA identification.

High throughput sequencing generates small RNA reads of 50 nucleotides in length (single reads). Raw reads were trimmed to remove adapter sequences using cutadapt software (version 1.4.1) [53]. For downstream analysis, only reads having length between 15 and 40 bases were considered.

### miRNA genes annotation

miRDeep2 algorithm [18] was used to detect miRNA from small RNA deep sequencing data. This algorithm uses a probabilistic model to score the fit of sequenced RNA to the biological model of miRNA biogenesis [17]. Briefly, reads are aligned to the *S. frugiperda* genome (Corn variant assembly version 3.1 or Rice variant assembly version 1.0), and only reads that do not map more than five times to the genome were used for miRNA detection. Then, using the read mappings as guidelines, potential miRNA precursors are excised from the genome and the miRDeep2 core algorithm scores their likelihood to be a real miRNA precursor. The output is a scored list of known and novel miRNA in the deep sequenced sample. Known miRNAs were identified by similarity to miRNA sequences from miRBase database (release 21).

### Orthology analysis

To explore the conservation of miRNAs between C and R strains, a reciprocal blastn was performed to search for orthologs [54, 55]. BLAST for miRNA mature sequences was run with blastn with default parameters except for a lower e-value threshold of 1e-3.

### Analysis of differential miRNA expression

miRDeep2 algorithm also provides read counts for the detected miRNA. To assess changes in miRNA expression between *S. frugiperda* variants and host-plant conditions, the read counts data for known and novel miRNA were used as input for the R package DESeq2 [56]. DESeq2 uses negative binomial generalized linear models to test for differential expression. An adjusted *p*-value for multiple testing was computed with the Benjamini-Hochberg procedure to control false discovery rate (FDR). Results with a FDR < 0.05 were considered statistically significant.

### Experimental validation of differential expression

The miRNA expression levels were quantified using TaqMan small RNA assay system from Life Technologies. Briefly, total RNA from samples was isolated using Trizol. One to 10 ng of total RNA was used for reverse transcription using a specific RT primer with the following conditions, i.e., 16 °C: 30 min, 42 °C: 30 min, 85 °C: 5 min. Subsequently, the cDNA was used for qRT analysis with TaqMan probes according to the manufacturer’s instructions. For all qRT based TaqMan assays, the qRT-PCR quantifications were performed on two biological replicates (pools of 12 larvae) of the reciprocal transplant experiments, with 3 technical replicates. During the qRT analysis, the 2-ΔΔCT method was employed and each Ct value of the test miR was normalized to that of an endogenous miRNA (*Sf* mir-31 5p) whose expression remained stable in the different experimental conditions that were tested in C and R strain. To check that the miR expression level differed significantly between two experimental conditions, we used a pair wise fixed reallocation randomization statistical test [57] (2000 iterations, p-value< 0.001) which avoids making any assumptions about distributions compared to standard parametric tests such as analysis of variance or t-tests.

### Detection and functional annotation of potential target genes regulated by miRNA

TargetScan [58–60] predicts biological targets of miRNAs by searching for the presence of conserved 8-mer and 7-mer sites that match seed region of each miRNA by calculating thermodynamic free energy using the RNAFold package [61]. Predictions are ranked using the site number, site type, and site context. TargetScan (version 5.0) was run with default parameters using mature known miRNAs and the 3’UTR of predicted coding genes set OGS2.2_UTR3 of the C strain available on the webportal [62]. Predictions obtained by TargetScan were confirmed by at least one of the three following softwares: MiRanda, Rna22 and miRmap. MiRanda (version v3.3a) [63] allows one wobble pairing in the seed region when it is compensated by matches in the 3′ end of the miRNA, it calculates the binding energy of the duplex structure and its position within the 3’UTR, it was used with the same parameters as in [64]. Rna22 (version v2) [65] is a tool based on a search for patterns that are statistically significant miRNA motifs created after a sequence analysis of known mature miRNAs, it was used with default parameters. MiRmap [66] is a web-based application [67] that combines many thermodynamic, evolutionary, probabilistic and sequence-based features.

## Additional files

Additional file 1: **Table S1.** Number of sequence reads in each small non coding RNAs library. (DOCX 14 kb)

Additional file 2: **Excel file S1.** Predictions of miR genes by mirDEEP2 in SfC and SfR and orthology table. (XLSX 92 kb)

Additional file 3: **Figure S1.** MA-plots showing the relative expression of known or novel miR according to the host-plant (Rice compared to corn) in each strain. Top panel, in SfC, bottom panel In SfR. (PPTX 5002 kb)

Additional file 4: **Figure S2.** Variation between samples (treatments, replicates) of larvae exposed to different plants displayed by Principal Component Analysis (PCA). Top panel: SfC, bottom panel: SfR. (PPTX 4001 kb)

Additional file 5: **Excel file S2.** Relative expression resulting from DESEQ2 analysis within strains according to the host-plant. In red novel miR, in black known miRs. (XLSX 53 kb)

Additional file 6: **Figure S3.** MA-plots showing the relative expression of known or novel miR according to the genetic background. Top panel, relative expression analyzed by DESEQ2 in SfR compared to SfC on corn, bottom panel, relative expression in SfR compared to SfC on rice. (PPTX 4997 kb)

Additional file 7: **Figure S4.** Variation between samples (treatments, replicates) of larvae exposed to different plants displayed by Principal Component Analysis (PCA). Top panel: SfR compared to SfC on corn, bottom panel: SfR compared to SfC on rice. (PPTX 3008 kb)

Additional file 8: **Excel file S3.** Relative expression resulting from DESEQ2 analysis according to the genetic background on the same host-plant. In red novel miR, in black known miRs. (XLSX 39 kb)

Additional file 9: **Excel file S4.** List of gene target predictions of known differentially expressed miRs. (XLSX 1578 kb)

Additional file 10: **Excel file S5.** Target genes of known miRs and their relative expression in SfC on rice compared to corn. (XLSX 35 kb)

Additional file 11: **Excel file S6.** Target genes of known miRs and their relative expression in SfR compared to SfC on corn. (XLSX 19 kb)
