# Supplementary material for: Characterization and expression profiling of microRNAs in response to plant feeding in two host-plant strains of the lepidopteran pest Spodoptera frugiperda
Source: BMC Genomics. 2018 Nov 6;19:804. doi: 10.1186/s12864-018-5119-6 (PMC6219076; doi:10.1186/s12864-018-5119-6)
Supplement: Supplementary file 1 — Table S1. Number of sequence reads in each small non coding RNAs library. (DOCX 14 kb) [file 12864_2018_5119_MOESM1_ESM.docx]

| Samples | CB* | CAF* | % (/CB) | Unique reads |
| --- | --- | --- | --- | --- |
| CC1 | 59 991 024 | 57 064 333 | 95.12 | 2 381 485 |
| CC2 | 40 919 195 | 38 883 923 | 95.03 | 3 019 566 |
| RC1 | 33 723 132 | 32 234 935 | 95.59 | 2 418 661 |
| RC2 | 33 137 701 | 31 556 641 | 95.23 | 2 039 941 |
| CR1 | 38 259 103 | 36 155 709 | 94.50 | 4 432 878 |
| CR2 | 40 969 752 | 38 548 824 | 94.09 | 5 363 838 |
| RR1 | 49 365 175 | 46 593 092 | 94.38 | 5 616 637 |
| RR2 | 45 691 315 | 43 336 005 | 94.85 | 4 462 848 |

*CB : Number of raw clusters

*CAF : Number of clusters after filtering and adapter trimming
