# Supplementary figures and images for: Characterization and expression profiling of microRNAs in response to plant feeding in two host-plant strains of the lepidopteran pest Spodoptera frugiperda
Source: BMC Genomics. 2018 Nov 6;19:804. doi: 10.1186/s12864-018-5119-6 (PMC6219076; doi:10.1186/s12864-018-5119-6)

## Slide 1
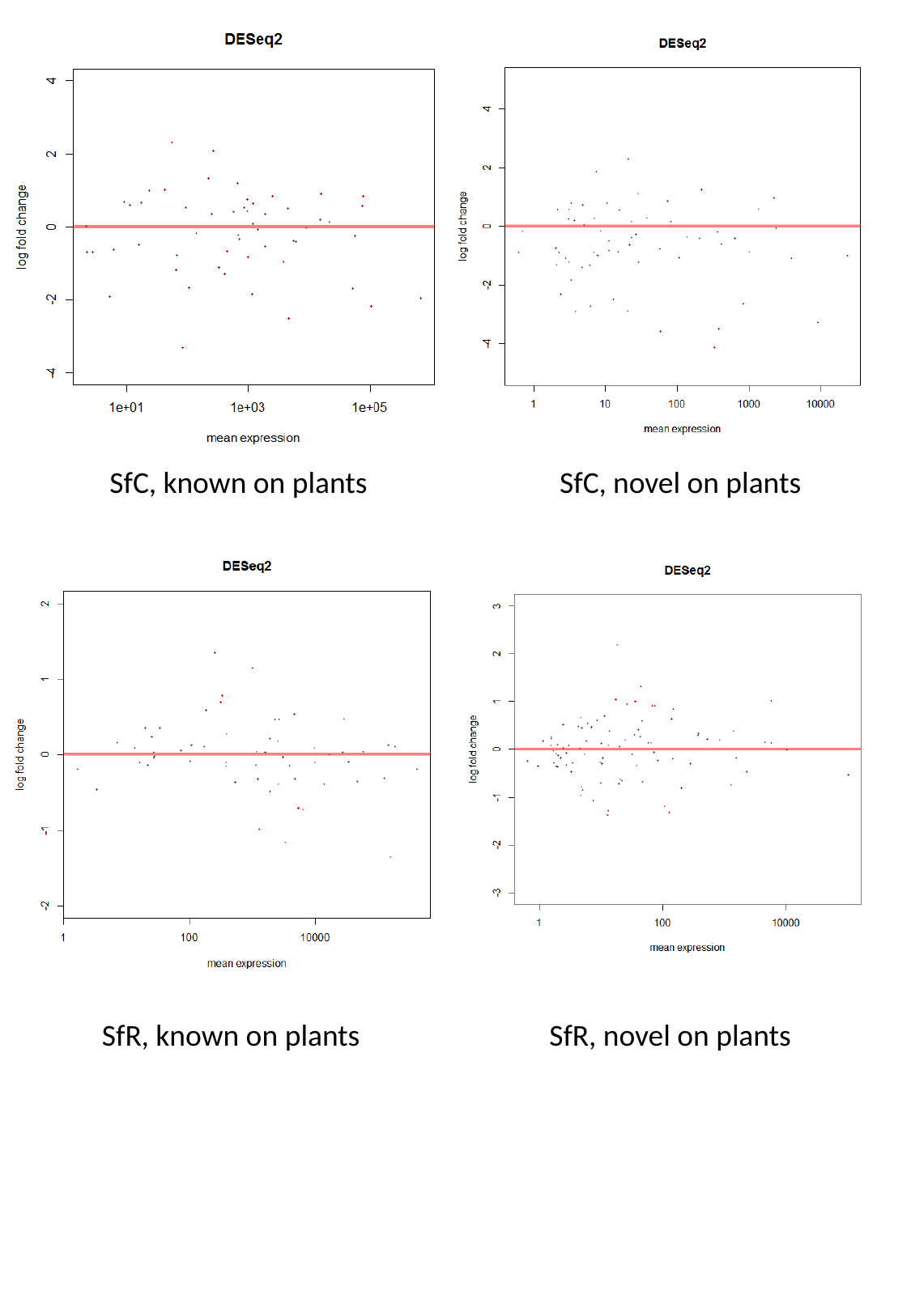

SfC, known on plants
SfC, novel on plants
SfR, known on plants
SfR, novel on plants

Supplement: Supplementary file 3 — Figure S1. MA-plots showing the relative expression of known or novel miR according to the host-plant (Rice compared to corn) in each strain. Top panel, in SfC, bottom panel In SfR. (PPTX 5002 kb) [file 12864_2018_5119_MOESM3_ESM.pptx]

## Slide 1
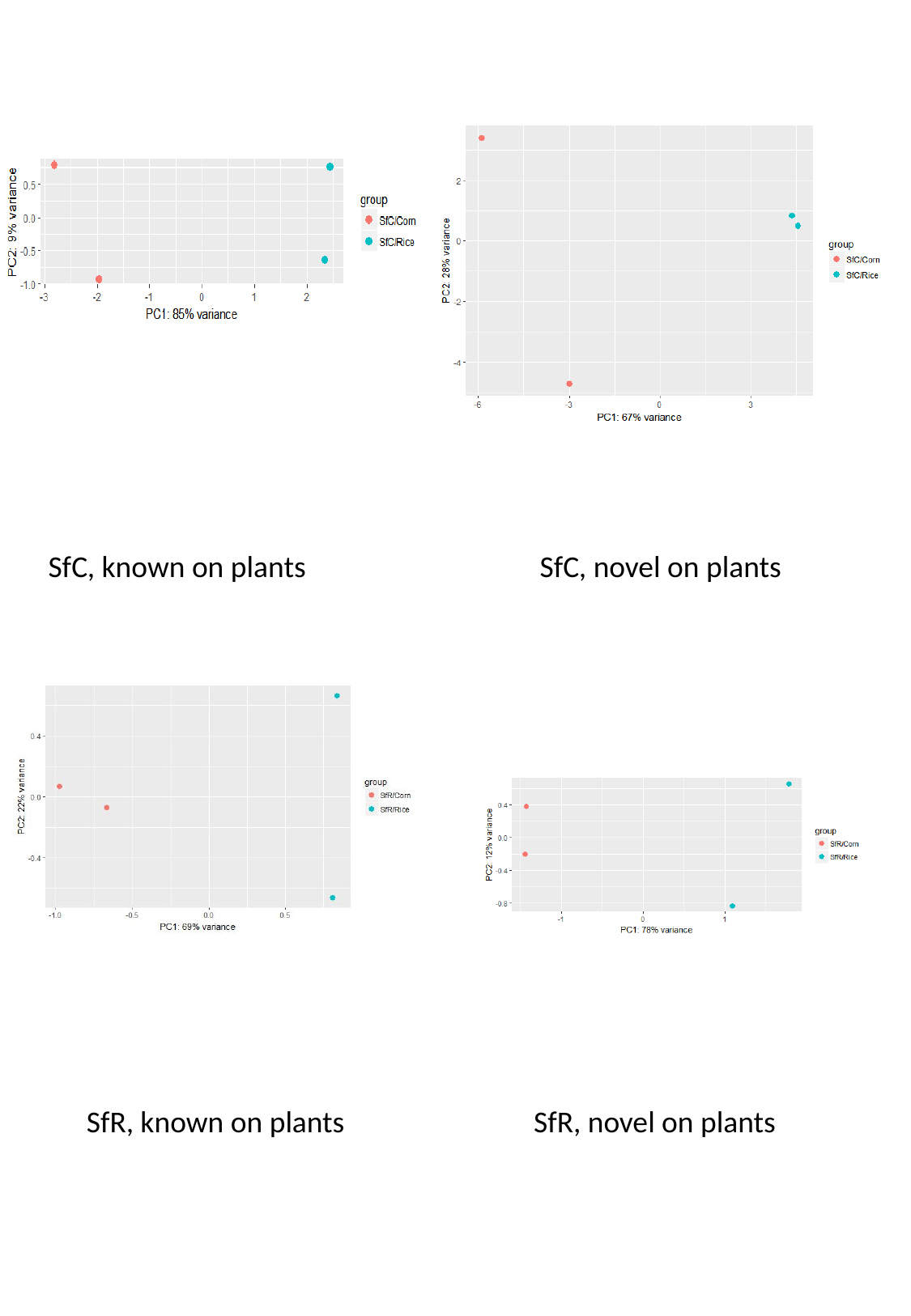

SfC, known on plants
SfC, novel on plants
SfR, known on plants
SfR, novel on plants

Supplement: Supplementary file 4 — Figure S2. Variation between samples (treatments, replicates) of larvae exposed to different plants displayed by Principal Component Analysis (PCA). Top panel: SfC, bottom panel: SfR. (PPTX 4001 kb) [file 12864_2018_5119_MOESM4_ESM.pptx]
