# Supplementary material for: Characterization and expression profiling of microRNAs in response to plant feeding in two host-plant strains of the lepidopteran pest Spodoptera frugiperda
Source: BMC Genomics. 2018 Nov 6;19:804. doi: 10.1186/s12864-018-5119-6 (PMC6219076; doi:10.1186/s12864-018-5119-6)
Supplement: Supplementary file 6 — Figure S3. MA-plots showing the relative expression of known or novel miR according to the genetic background. Top panel, relative expression analyzed by DESEQ2 in SfR compared to SfC on corn, bottom panel, relative expression in SfR compared to SfC on rice. (PPTX 4997 kb) [file 12864_2018_5119_MOESM6_ESM.pptx]

## Slide 1
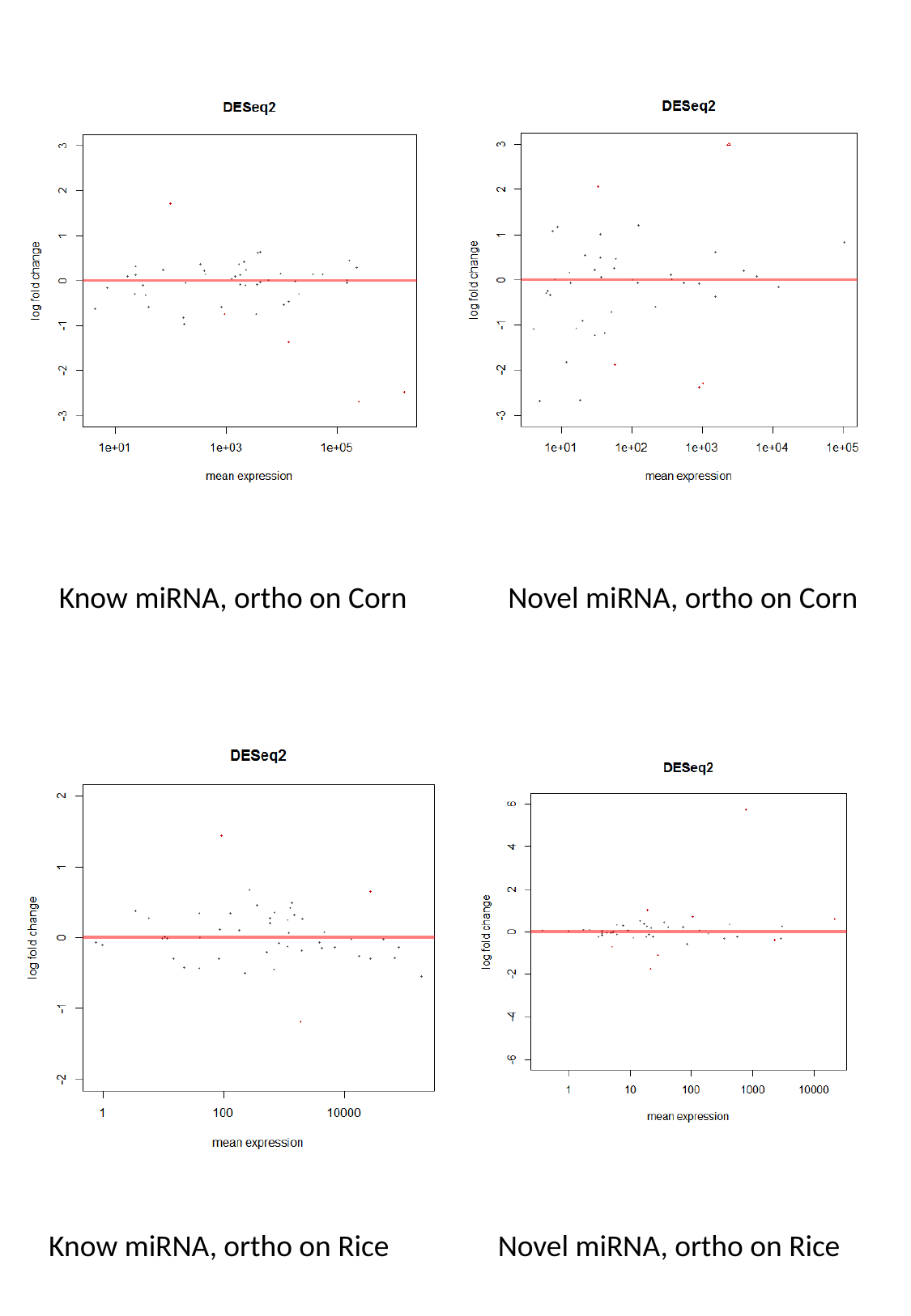

Know miRNA, ortho on Corn
Novel miRNA, ortho on Corn
Know miRNA, ortho on Rice
Novel miRNA, ortho on Rice
