# Supplementary material for: Characterization and expression profiling of microRNAs in response to plant feeding in two host-plant strains of the lepidopteran pest Spodoptera frugiperda
Source: BMC Genomics. 2018 Nov 6;19:804. doi: 10.1186/s12864-018-5119-6 (PMC6219076; doi:10.1186/s12864-018-5119-6)
Supplement: Supplementary file 7 — Figure S4. Variation between samples (treatments, replicates) of larvae exposed to different plants displayed by Principal Component Analysis (PCA). Top panel: SfR compared to SfC on corn, bottom panel: SfR compared to SfC on rice. (PPTX 3008 kb) [file 12864_2018_5119_MOESM7_ESM.pptx]

## Slide 1
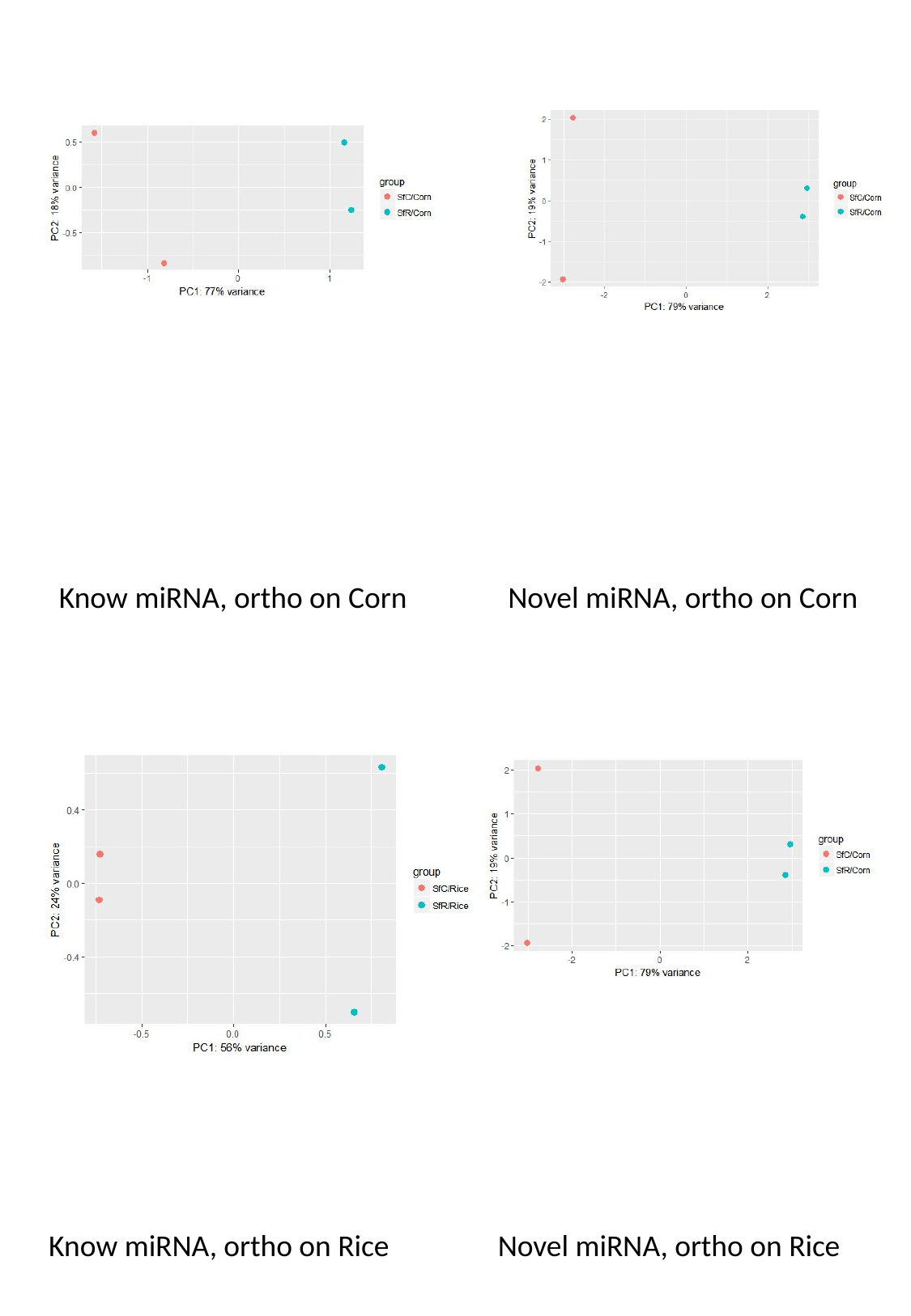

Know miRNA, ortho on Corn
Novel miRNA, ortho on Corn
Know miRNA, ortho on Rice
Novel miRNA, ortho on Rice
